# Impact of radiation attenuation by a carbon fiber couch on patient dose verification

**DOI:** 10.1038/srep43336

**Published:** 2017-02-27

**Authors:** Chun-Yen Yu, Wen-Tsae Chou, Yi-Jen Liao, Jeng-Hung Lee, Ji-An Liang, Shih-Ming Hsu

**Affiliations:** 1 Department of Biomedical Engineering and Environmental Sciences, National Tsing Hua University, Hsinchu, Taiwan, ROC; 2 Medical Physics and Radiation Measurements Laboratory, Department of Biomedical Imaging and Radiological Sciences, National Yang-Ming University, Taipei, Taiwan, ROC; 3 3Department of Biomedical Imaging and Radiological Science, China Medical University, Taichung, Taiwan, ROC; 4 School of Medical Laboratory Science and Biotechnology, Collage of Medical Science and Technology, Taipei Medical University, Taipei, Taiwan, ROC; 5 Health Physics Division, Institute of Nuclear Energy Research, Longtan, Taiwan, ROC; 6 Department of Radiation Oncology, China Medical University Hospital, Taichung, Taiwan, ROC; 7 Department of Biomedical Imaging and Radiological Sciences, National Yang-Ming University, Taipei, Taiwan, ROC; 8 Biophotonics and Molecular Imaging Research Center, National Yang-Ming University, Taipei, Taiwan, ROC

## Abstract

The aim of this study was to understand the difference between the measured and calculated irradiation attenuations obtained using two algorithms and to identify the influence of couch attenuation on patient dose verification. We performed eight tests of couch attenuation with two photon energies, two longitudinal couch positions, and two rail positions. The couch attenuation was determined using a radiation treatment planning system. The measured and calculated attenuations were compared. We also performed 12 verifications of head-and-neck and rectum cases by using a Delta phantom. The dose deviation (DD), distance to agreement (DTA), and gamma index of pencil-beam convolution (PBC) verifications were nearly the same. The agreement was least consistent for the anisotropic analytical algorithm (AAA) without the couch for the head-and-neck case, in which the DD, DTA, and gamma index were 74.4%, 99.3%, and 89%, respectively; for the rectum case, the corresponding values were 56.2%, 95.1%, and 92.4%. We suggest that dose verification should be performed using the following three metrics simultaneously: DD, DTA, and the gamma index.

The goal of radiation therapy is to deliver the prescribed dose to a tumor in a concentrated manner while minimizing the dose to normal organs. This aim is challenging because of movement uncertainties caused by patient respiration, positioning errors, and couch attenuation. Several decades ago, the support rails of treatment couches were made of steel, and they were associated with substantial radiation attenuation. The dose distribution in the patient’s body was unknown at that time, and the dose that penetrated the rails was not compensated for in the treatment plan. An incident gantry angle that avoided penetration of the support rails by the beam was calculated by using a sophisticated planning protocol. However, Buckle, by using a computer program, has predicted that the treatment field is also significantly attenuated by other parts of the couch^1^. In addition, the rails on both sides and in the middle of a couch can impede beam penetration. Unfortunately, a larger irradiation field will always result in radiation penetrating the rails, regardless of positioning.

A novel carbon fiber couch that is associated with little radiation attenuation was designed 1–2 decades ago. Numerous investigators have studied the attenuation of this carbon fiber couch. McCormack has found that a 6-MV photon beam is attenuated by 9% for a gantry angle of 70°^2^. Vieira has measured the attenuation of the carbon fiber couch with an electronic portal imaging device (EPID) for the case of intensity-modulated radiation therapy (IMRT) fields and has found a couch attenuation as high as 15% for a 6-MV photon beam^3^. Moreover, Li has found the couch attenuation to be as high as 25.2% with a 6-MV photon beam^4^. Poppe has reported a 6-MV beam attenuated by 2.7% to 6.4% and a 10-MV beam attenuated by 2.3% to 4.9% at an angle of 150° with a Primus Digital Mevatron linear accelerator (Siemens Medical Solutions, Concord, CA, USA)^5^. Wanger has also measured the couch attenuation with a cylindrical phantom with 6-MV and 20-MV photon beams^6^,^7^. Wanger has found that the attenuation due to the support rail ranges from 8.83% to 17.01%. Wanger has also determined the difference between the measured and calculated attenuations by using the anisotropic analytical algorithm (AAA) in a radiation treatment planning (RTP) system; in this system, the difference was minimized by use of assigned attenuation values of −750 Hounsfield units (HU) for the carbon plate, −995 HU for the carbon plate filling, and 225 HU for the rails.

In the present study, two algorithms—pencil-beam convolution (PBC) and the AAA—were applied with an Eclipse RTP system (version 11.0, Varian Medical Systems, Palo Alto, CA, USA). The treatment plans were calculated with the AAA, and the patients were treated with RapidArc. Another group of patients was treated with IMRT or three-dimensional conformal radiotherapy. Their treatment plans were executed with PBC or the AAA, depending on which linear accelerator was used. Some authors have suggested that the couch attenuation should be accounted for during treatment calculations and the actual irradiation protocol, particularly for posterior beams. Therefore, to understand the difference between measured and calculated attenuations obtained by using two algorithms and to determine the influence of couch attenuation on patient dose verification, we measured the attenuation at various gantry angles and photon energies and verified the doses received by the patients.

## Results

**Figure 1:**
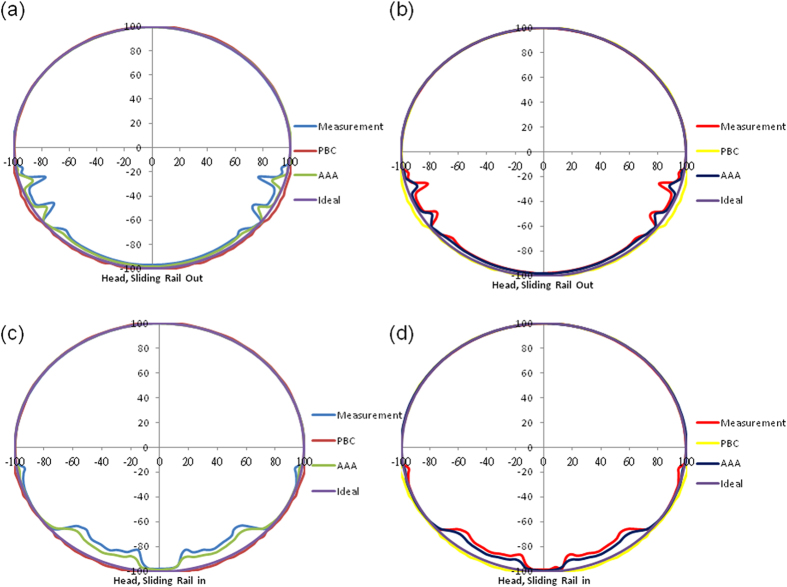
Measured transmission values and those calculated using PBC and the AAA at the head position for irradiation at (**a**) 6 MV with rails on both sides, (**b**) 10 MV with rails on both sides, (**c**) 6 MV with rails in the middle of the couch, and (**d**) 10 MV with rails in the middle of the couch. The dashed line is an ideal circle with a radius of 1 unit.

Figure 1 shows the measured and calculated transmission values for the bullet phantom at the head position. The transmission was measured as the length at the specified angle. For irradiation at 6 MV with rails on both sides of the couch, the measured transmission values were markedly lower than 1 unit in the following three sections: (1) at beam angles from 100° to 125° (IEC 1217 scale), for which the maximum attenuation was 19.5%, (2) at beam angles from 135° to 225°, which penetrated the central spine of the head position of the couch^3^, for which the attenuation was approximately 4%, and (3) at beam angles from 235° to 260°, for which the maximum attenuation was 19.5%. The beam angles in the first and third sections penetrated the rails. In these three sections, the transmission values calculated with PBC were larger than 1 unit. Relative to PBC, the transmission values with the AAA were lower than 1 unit and closer to the measured transmission values in the three sections (Fig. 1a).

The transmission values for irradiation at 10 MV were much lower than 1 unit in the same sections as those described for irradiation at 6 MV (Fig. 1b). The maximum attenuations in the first and third sections were 15.1% and 14.8%, respectively, whereas the attenuation in the second section was approximately 2.5%. When the rails were in the middle of the couch, the transmission values were much lower than 1 unit for angles of approximately 100° in the first section, from 137.5° to 172.5° in the second, from 187.5° to 222.5° in the third, and approximately 260° in the fourth. For irradiation at 6 MV, the attenuations in the four sections were approximately 5%, 14%, 13.5%, and 5%, respectively (Fig. 1c); for irradiation at 10 MV, the corresponding attenuations were approximately 4%, 10%, 9.5%, and 4% (Fig. 1d).

**Figure 2:**
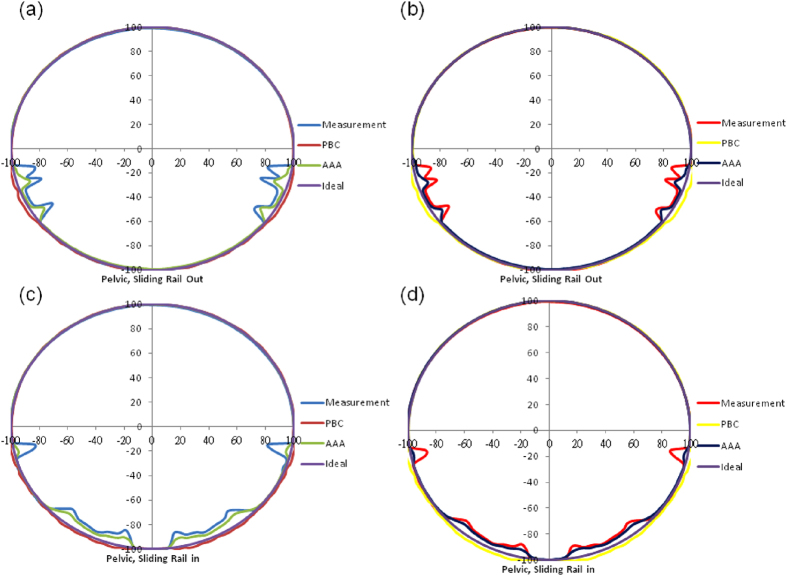
(**a–d**) Same as Fig. 1, but at the pelvis position. A_L_, B_L_, C_L_, A_R_, B_R_, and C_R_ at angles of 100°, 107.5°, 122.5°, 237.5°, 252.5°, and 260°, respectively, represent the largest attenuations.

With the bullet phantom at the pelvis position and rails on both sides, the transmission values were much lower than 1 unit in two sections. The angles in these two sections were the same as those in the first and third sections of the bullet phantom at the head position. The maximum attenuations in the two sections were 19.5% and 17.8% for irradiation at 6 MV (Fig. 2a) and 14.8% and 13.7% for irradiation at 10 MV (Fig. 2b). When the rails were in the middle of the couch, the angles in the four sections were the same as those with the bullet phantom at the head position. The attenuations in sections 1 to 4 were approximately 16.5%, 9%, 8.5%, and 15.7%, respectively, for irradiation at 6 MV (Fig. 2c), and approximately 12.6%, 6.8%, 6.3%, and 11.4%, respectively, for irradiation at 10 MV (Fig. 2d). For the phantom at the pelvis position, the attenuation calculated with PBC remained larger than the measured attenuation in all sections. The attenuations calculated with the AAA were much closer to the measured values than the attenuations calculated with PBC.

The values of the three metrics (dose deviation (DD), distance to agreement (DTA), and the gamma index) in the six verifications for the head-and-neck case are shown in Table 1. The tolerances of DD, DTA, and the gamma index were 3% in percentage dose, 3 mm in distance, and 1 unit, respectively. The pass ratio of the gamma index was 90%. The DDs for PBC_G0, PBC − Couch, and PBC + Couch were 88.7%, 88.5%, and 88.1%, respectively; the corresponding values for the AAA verifications were 94.8%, 74.4%, and 90.6%. The DDs of the three PBC verifications were nearly the same, even for PBC − Couch. The DD for AAA − Couch was markedly different from the values for AAA_G0 and AAA + Couch. We also found that the gamma index of the three PBC verifications differed only slightly. However, the gamma index for AAA − Couch differed markedly from the values for AAA_G0 and AAA + Couch.

The other six verifications for the rectum case are shown in Table 2. The DDs for PBC_G0, PBC − Couch, and PBC + Couch were 72.5%, 71.4%, and 69.6%, respectively; the corresponding values for the AAA were 82%, 56.2%, and 82%. Because the rectum is relatively small and close to critical organs, such as the bladder and rectum, the DDs of PBC and the AAA were much lower than those of the head-and-neck case. The DD and gamma index for AAA − Couch differed substantially from the values for AAA_G0 and AAA + Couch; in contrast, the DDs of PBC differed only slightly. All verification metrics of the AAA had better agreement than those of PBC, thus suggesting that the calculations of the AAA are more precise than those of PBC.

**Figure 3:**
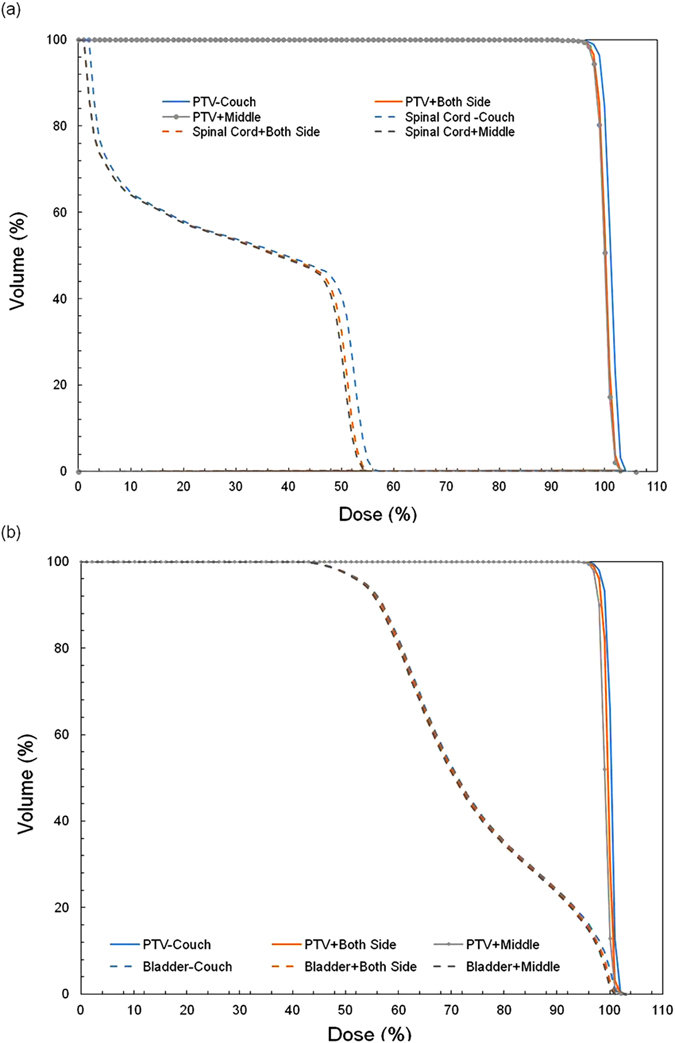
(**a**) DVH of head-and-neck case. The solid blue line represents the DVH of PTV in the treatment plan without the couch. The dashed blue line represents the DVH of the spinal cord without the couch. The solid orange line represents the DVH of PTV in the treatment plan with the rail on both sides. The dashed orange line represents the DVH of the spinal cord with the rail on both sides. The solid gray line represents the DVH of PTV in the treatment plan with the rail in the middle of the couch. The dashed gray line represents the DVH of the spinal cord with the rail in the middle of the couch. (**b**) DVH of the rectum case. The solid blue line represents the DVH of PTV in the treatment plan without the couch. The dashed blue line represents the DVH of the bladder without the couch. The solid orange line represents the DVH of PTV in the treatment plan with the rail on both sides. The dashed orange line represents the DVH of the bladder with the rail on both sides. The solid gray line represents the DVH of PTV in the treatment plan with the rail in the middle of the couch. The dashed gray line represents the DVH of bladder with the rail in the middle of the couch.

Figure 3 presents the effect of the absence of the couch in the treatment plan on patient dose for head-and-neck and rectum cases. In Fig. 3a, the D95 of PTV is 99.1%, 98.2% and 97.8% for PTV − Couch, PTV + Both sides and PTV + Middle, respectively. For D5 of the spinal cord, the difference between PTV − Couch and PTV + Both sides is 1.0% (53.9%–52.9%), and the difference between PTV − Couch and PTV + Middle is 2.1% (53.9%–51.8%). In Fig. 3b, the D95 of PTV is 98.8%, 98.1% and 97.3% for PTV − Couch, PTV + Both sides and PTV + Middle, respectively. The dose difference in the bladder is less than 0.5%.

The accuracy of RapidArc verifications for both cases is shown in Table 3. The agreement was better when the rail was configured to be in the same position in the plan and measurements. The dose deviation was 4.14% and 3.66% for the head-and-neck and rectum case, respectively. We determined that for both cases. when the couch was absent from the treatment plan and the rail was configured on both sides, the results were somewhat worse than when the couch was present in the treatment plan and the rail was configured on both sides. We determined that the 169.9 cGy dose at the center of the cubic phantom without the couch was reduced to 168.8 cGy when the rail was positioned on both sides and to 167.5 cGy when the rail was positioned in the middle of the couch during calculations for the head-and-neck case. The same conclusions pertain to the rectum case.

## Discussion

**Figure 4:**
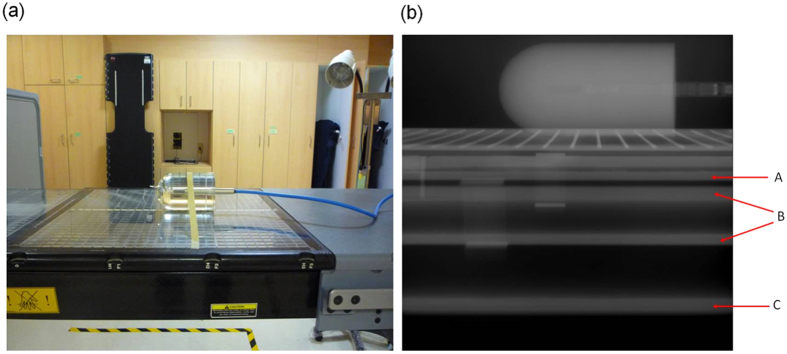
(**a**) The ion chamber was positioned at a source-axis distance of 100 cm with a buildup cap. The 3.7-cm radius of the buildup cap was larger than the dmax values of 6 MV and 10 MV to ensure electron equilibrium. (**b**) Electronic portal imaging for a beam angle of 270°. The first gray bar, labeled A, represents the edge of the couch plate. The second and third bars, labeled B, represent the upper edge of the rail. The fourth bar, labeled C, represents the lower edge of the rail.

Our data indicate that the rails markedly attenuated the radiation, by as much as 19.5% for radiation at 6 MV and 15.1% for radiation at 10 MV. Li has found that rails produced attenuations of up to 25.2% and 14.4% for radiation at 6 MV and 10 MV, respectively^4^. The maximum attenuation found by Wagner was 17.1%^6^. In addition to the rails, the edges of the couch plate also substantially attenuated the radiation. The attenuation of radiation by the edges of the couch plate and rails was demonstrated with an EPID (Fig. 4, at a gantry angle of 270°). In Fig. 4b, the gray bar labeled A represents the edges of the couch plate, B represents the upper edge of the couch rail, and C represents the lower edge of the couch rail. It is straightforward to determine the six largest attenuations for the bullet phantom at the pelvis position with rails on both sides (Fig. 2a and b). The six largest attenuations are labeled A_L_, B_L_, C_L_, A_R_, B_R_, and C_R_, at angles of 100°, 107.5°, 122.5°, 237.5°, 252.5°, and 260°, respectively. A_L_ and A_R_ coincide with the edges of the couch plate, B_L_ and B_R_ coincide with the upper edge of the left and right rails, and C_L_ and C_R_ coincide with the lower edge of the left and right rails. We also observed that the six largest attenuations for the bullet phantom at the head position with rails on both sides were located at the same angle as those for the phantom at the pelvis position. However, the small attenuations caused by the edges of the couch plate were 4.9% at 100° and 4.9% at 260° for radiation at 6 MV and only 3.5% and 3.6% for radiation at 10 MV. These differences are difficult to visualize in Fig. 2b.

The largest difference between PBC calculations and the measured values was 23.1% at 107.5° with 6 MV and 19.9% at 252.5° with 10 MV. The largest difference between the AAA − derived calculations and the measured values was 9.8% at 107.5° with 6 MV and 10.2% at 100° with 10 MV. The six lowest transmission values are listed in Table 4. The AAA − derived calculations were much closer than the PBC − derived calculations to the measured values.

The PBC calculations indicated that the beam penetrated the couch without any attenuation and penetrated the rails. An alternative explanation is that the contribution of the scattering of the dose from the rails to the phantom was greater than the attenuation of the primary dose. Therefore, we question the validity of the PBC calculations, particularly for the posterior beam.

Because the PBC − calculated transmission values were so different from the measurements, we initially predicted that the verification of PBC − Couch would yield poor DD, DAT, and gamma index values. However, the results obtained for PBC_G0, PBC − Couch, and PBC + Couch did not support this conclusion for either case—the DD, DAT, and gamma index for PBC − Couch were nearly the same as the values for PBC_G0 and PBC + Couch. There was also no clear difference in DAT between the three AAA − derived verifications. However, the DD and gamma index for AAA − Couch was markedly different from the values for AAA_G0 and AAA + Couch. This finding suggests that the attenuation of the couch rails substantially affected the accuracy of the AAA verifications.

The agreement between the AAA + Couch values for both cases was nearly the same as that for AAA_G0, thus indicating that the couch attenuation was precisely calculated with the AAA. Although the agreement of PBC + Couch values for both cases differed only slightly from that for PBC_G0, we cannot conclude that the couch attenuation was precisely calculated with PBC. The agreement of PBC − Couch for both cases was similar to that for PBC_G0 and PBC + Couch. Although the couches were absent in PBC − Couch and present in PBC + Couch, the results for PBC − Couch and PBC + Couch were indistinguishable. Therefore, we cannot conclude that the couch attenuation was precisely calculated with PBC.

The values of the three metrics for AAA_G0 and AAA + Couch demonstrated better agreement than those for PBC − G0 and PBC + Couch, thus demonstrating that the AAA is more precise. However, the verification parameters for AAA − Couch for both cases demonstrated less agreement than those for PBC − Couch. Because the AAA is more precise, a defect would result in a greater disagreement than for PBC. Therefore, the DD and gamma index for AAA − Couch demonstrated lower agreement than for PBC − Couch.

The gantry angle from 137.5° to 172.5° and from 187.5° to 222.5° may always be attenuated approximately 4% when the rail is configured in the middle of the couch. It is difficult to avoid the specified gantry angle, even for a small field. The incident beam easily penetrated both the upper edge and lower edge of the rail, thus implying that the attenuation will be greater, and positioning the rail in the middle of the couch reduced the dose certainty more than positioning the rail on both sides.

The rail position changed not only the dose in the target but also that in normal organs. In the head-and-neck case, the dose for the spinal cord for +Both sides and +Middle plan was less than −Couch, but the dose in the bladder was nearly identical across the three plans. In addition, the beam incident on the patient’s back may penetrate the spinal cord before the target for head-and-neck. For the rectum, the back beam may penetrate the target before the bladder. Positioning the rail at the middle of the couch may be inappropriate for a tumor of the central body, such as spine metastasis. The tumor will receive a lower dose, and tumor coverage will be reduced. It may also be inappropriate to position the rail on both sides for tumors on the body side.

## Conclusion

The results obtained in this study demonstrate that the AAA is more precise than PBC and that the AAA is therefore more suitable for attenuation calculations. Moreover, most couch attenuation was due to the couch rail and the edge of the couch plate. Couch attenuation and rail position should be accounted for during treatment calculations and verification.

## Methods

### Couch attenuation measurement

Couch attenuation was measured for photon energies of 6 MV and 10 MV with a Varian 21iX linear accelerator (Varian Medical Systems, USA). The linear accelerator couch has two translatable rails and a grid couch insert, all of which are constructed from carbon fiber. The rails can be parked on both sides or in the middle of the couch. Two longitudinal positions, the head and pelvis, are present in a couch. An acrylic bullet phantom was positioned at the middle of the couch. The diameter of the round end of the bullet phantom was 7.4 cm, and its total height was 12.7 cm. An ion chamber (TN30013, Farmer chamber, PTW, Freiburg, Germany) was inserted into the bullet phantom at a source-axis distance of 100 cm (Fig. 4a), and the phantom dose was measured for gantry angles from 0° to 360° (IEC 1217 scale). Measurements were made every 5° for anterior gantry angles and every 2.5° for posterior gantry angles. The average value of the doses measured at gantry angles of 0°, 90°, and 270° was used as a reference.

We performed eight tests of the couch attenuation with two photon energies, two longitudinal positions, and two rail positions. All measurements were made with an irradiation field size of 10 × 10 cm^2^ and with the linear accelerator operating at 50 MU.

**Figure 5:**
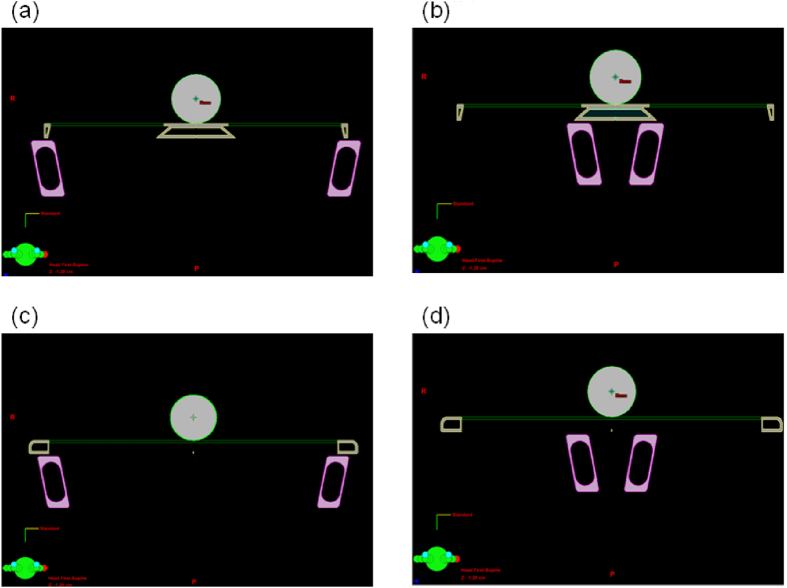
Transmission values for irradiation at 6 MV and 10 MV calculated under various conditions, with the phantom placed (**a**) at the head position with rails on both sides, (**b**) at the head position with rails in the middle of the couch, (**c**) at the pelvis position with rails on both sides, or (**d**) at the pelvis position with rails in the middle of the couch.

To calculate the couch attenuation, a virtual couch and a virtual phantom (which had a diameter of 7.4 cm and a height of 12.7 cm) were created for treatment planning. HU was applied to the phantom using 112. For the couch in the planning system, the manufacturer’s default settings of −300 HU for the couch plate, −1000 HU for the foam inside the carbon plate, and 200 HU for the rails were used. Both the virtual couch and phantom were combined as a “body” because the voxels in the body could be calculated with only PBC. All calculated situations in which the virtual phantom was irradiated in the RTP were the same as in the experimental measurements (Fig. 5).

One head-and-neck case irradiated at 6 MV and one rectum case irradiated at 10 MV were used for patient dose verification. For both cases, the rails were parked on both sides of the couch. Seven beams were applied at angles of 0°, 50°, 100°, 140°, 220°, 260°, and 310° for the head-and-neck case and angles of 30°, 80°, 130°, 180°, 230°, 280°, and 330° for the rectum case. We adjusted the incident angle for the head-and-neck case and ensured that those at 140° and 220° penetrated the rails during the measurements and in the treatment calculations. Six verification plans were created with a Delta phantom (Scandidos, Uppsala, Sweden) for each case. Both the virtual couch and phantom were combined into a single body.

### Treatment planning system

#### IMRT

Three plans were calculated with PBC, whereas the others were calculated with the AAA. All seven beam angles were set at 0° in one of the three plans calculated with PBC; this verification plan was denoted PBC_G0. In another verification plan (denoted PBC − Couch), the seven beam angles irradiating the patient for treatment were calculated without the couch. We sought to determine the agreement of the verification without the couch in the plan. The seven beam angles were the same for treatment of the patient in the other verification plan. The plan denoted by PBC + Couch was calculated with the couch. For PBC, three analogous verification plans were created and calculated with the AAA: AAA_G0, AAA − Couch, and AAA + Couch. The verifications of PBC_G0 and AAA_G0 represent standards of penetration occurring in the absence of rails during the measurements and treatment calculations.

#### RapidArc

The ability of treatment planning system (TPS) dose calculation algorithms to consider these devices is either lacking or rarely implemented by the user7. The two cases were planned with RapidArc; the couch was absent in the image during calculation. Two other verification plans were created from the same patient’s CT images. The rail in the middle of the couch was configured in the patient’s CT image for one of the verification plans. The PTV and normal organs of three plans were compared in a DVH.

### Dose verification

#### IMRT

The dose in the six verification plans was verified with the Delta^4^ phantom in the treatment room. We arranged 1,069 p-type silicon diodes on the main plane and two wing planes in the Delta phantom. The three planes were visualized as cross-penetrating a cylindrical polymethylmethacrylate phantom with a diameter of 22 cm and height of 40 cm. Each diode had an area of 0.0078 cm^2^, and they were spaced at 0.5-cm intervals over the central 6 × 6 cm^2^ area of the planes and at 1-cm intervals over the remainder of the central 20 × 20 cm^2^ area of the planes^8^. The measurements from the Delta phantom and calculations of the verification plan were analyzed on the basis of the DD, DTA, and gamma index^9^,^10^.

DD was computed, point-by-point, using local spatial interpolation, if necessary, to co-locate the evaluated dose distribution and the reference distribution. DTA is the distance between a reference data point and the nearest point in the compared dose distribution that exhibits the same dose. The gamma index was set to less than 1.

#### RapidArc

Three verification plans were created, which were based on each RapidArc treatment plan for the head-and-neck case. A solid water cubic phantom, 15 cm high, 30 cm wide, and 30 cm long, was applied in the verification plans. Three analogous verification plans were created for the rectum case. The solid water cubic phantom was also applied in the plans. The total MU was 401. During the measurements, the ion chamber (TN30013, Farmer chamber, PTW, Freiburg, Germany) was positioned at the geometric center of the cubic phantom.

## Additional Information

**How to cite this article**: Yu, C.-Y. *et al*. Impact of radiation attenuation by a carbon fiber couch on patient dose verification. *Sci. Rep.* **7**, 43336; doi: 10.1038/srep43336 (2017).

**Publisher's note:** Springer Nature remains neutral with regard to jurisdictional claims in published maps and institutional affiliations.

## Figures and Tables

**Table 1:** Agreement of the six verifications for the head-and-neck case.

	PBC(G0)	PBC − Couch	PBC + Couch	AAA(G0)	AAA − Couch	AAA + Couch
DD (%)	88.7	88.5	88.1	94.8	74.4	90.7
DTA (%)	96.6	98.6	95.9	100	99.3	100
γ index (%)	98.7	96.2	95.3	99.6	89	96.8

**Table 2:** Agreement of the six verifications for the rectum case.

	PBC(G0)	PBC − Couch	PBC + Couch	AAA(G0)	AAA − Couch	AAA + Couch
DD	72.5	71.4	69.6	82	56.2	82
DTA	95.3	97.3	95.8	99.4	95.1	99.4
γ index	97.1	98.4	94.3	98	92.4	98

**Table 3:** Agreement of the RapidArc verifications for the head-and-neck and rectum cases.

Couch Position		Head-and-Neck (cGy)			Rectum (cGy)		
Calculation	Measurement	Plan	Measured	Deviation	Plan	Measured	Deviation
−couch	Both sides	169.9	166.0	2.35%	236.6	230.7	2.56%
−couch	Middle	169.9	163.9	3.66%	236.6	227.2	4.14%
+Both sides	Both sides	168.8	166.0	1.69%	235.7	230.7	2.17%
+Middle	Middle	167.5	163.9	2.20%	232.0	227.2	2.11%

−couch indicates plan calculations without the couch.  + Both sides indicates calculations with the rail on both sides.  + Middle indicates calculations with the rail in the middle of the couch.

**Table 4:** The six maximum attenuation values among measurements and treatment calculations with the rails on both sides.

Gantry	100°		107.5°		122.5°		237.5°		252.5°		260^0^	
Energy (MV)	6	10	6	10	6	10	6	10	6	10	6	10
Measurement	83.6	87.7	80.5	85.2	84.6	88.6	83.7	87.7	82.2	86.3	84.8	88.5
PBC	102.0	102.9	102.6	103.7	102.7	103.7	102.7	103.7	102.4	103.6	102.1	103.0
AAA	97.6	97.9	90.3	92.7	91.0	93.4	90.8	93.3	90.5	92.9	97.9	98.1
